# Polymorphism of the *ABO* gene associate with thrombosis risk in patients with paroxysmal nocturnal hemoglobinuria

**DOI:** 10.18632/oncotarget.21361

**Published:** 2017-09-28

**Authors:** Zhangbiao Long, Yali Du, Hongmin Li, Bing Han

**Affiliations:** ^1^ Department of Hematology, Peking Union Medical College Hospital, Chinese Academy of Medical Sciences and Peking Union Medical College, Beijing, 100730, China

**Keywords:** polymorphism, gene, thrombosis risk, paroxysmal nocturnal hemoglobinuria

## Abstract

Thrombosis is one of the most common causes of mortality in Paroxysmal nocturnal hemoglobinuria (PNH), but the predisposing factors for thrombosis are yet to be defined. In this study, we outline the clinical characters and the susceptible genes which lead to thrombotic formation in 104 patients with PNH. The results displayed that the genotypes with minor alleles of rs495828 or rs2519093 in the *ABO* gene were associated with high risk to thrombus formation (OR 5.95, 95% CI 1.90-18.65 and OR 6.3, 95% CI 2.01-19.79, respectively). Further, the TT haplotype was associated with a significant increased risk of thrombosis (OR=3.25, 95%CI 1.42-7.39). Multivariate regression analysis showed larger PNH clone and genotypes with rs495828/rs2519093 minor allele as independent risk factors for thrombosis in PNH. Some patients who came back for follow-up were tested for the plasma levels of vWF and factor VIII. Patients carrying the rs495828/rs2519093 minor allele had a significant higher level of vWF and factor VIII compared with those carrying the major allele. Therefore, we found for the first time that the rs495828/rs2519093 polymorphism represent an independent prognostic factor in PNH patients for thrombus formation, probably by increasing the vWF and factor VIII.

## INTRODUCTION

Paroxysmal nocturnal hemoglobinuria (PNH) is a rare disease caused by an acquired mutation of the X-linked *PIG-A* (phosphatidylinositol glycan class A) gene on the hematopoietic stem cell. The *PIG-A* mutation disturbs the biosynthesis of glycosylphosphatidylinositol (GPI) anchor, which is necessary to attach dozens of membrane proteins to the cell surface [1]. Deficiency of the GPI-anchored complement regulatory proteins CD55 and CD59 on blood cells (CD55 inhibits the C3 and C5 convertases, CD59 blocks the assembly of the membrane attack complex), results in more vulnerable to the action of complement, and therefore leading to complement-mediated lysis [2, 3].

The main clinical manifestations of PNH include hemolytic anemia, thrombosis and bone marrow failure. However, Thromboembolism is one of the most common causes of mortality in PNH and accounts for approximately 40% to 67% of deaths [4, 5]. The incidence of thrombotic in PNH patients is various among different ethnic groups, 29%-44% in Caucasians [6, 7],18% in Korean [8], whereas 11.5% in Chinese [9]. According to the data from the international PNH registry, the incidence is 15.5% [10]. However, Thrombosis in PNH frequently occurs in vital site, either in western or eastern countries, which cause severe complications or even death [11, 12].

Multiple mechanisms including platelet activation, free hemoglobin release, nitric oxide depletion, urokinase type plasminogen activator receptor absence and endothelial dysfunction contribute to thrombosis in PNH, nevertheless, the predisposing factors and mechanisms for thrombosis are yet to be clearly elucidated [13–15].

To further identify intrinsic risk factors which lead to thrombotic formation, twenty-six SNPs in 17 genes which had been reported as high risk factors for venous thromboembolism (VTE) were detected [16–18]. Subsequently the correlation between clinical factors and thrombotic events was analyzed. The results showed the genotypes in the *ABO* gene were associated with high risk to thrombus formation,*ABO* gene is located at chromosome 9p 34.1-34.2, determines not only the biosynthesis of blood group antigen, but also encodes glycosyltransferases responses for post translational glycosylation of vWF, which protect vWF from proteolysis [19, 20]. As the carrier molecule of factor VIII, the vWF level further affect the level of factor VIII [21]. Therefore, patients who came back for follow-up were tested for the plasma levels of vWF and factor VIII, the risk factors associated with thromboembolism and the possible underlying mechanism was also investigated in this study.

## RESULTS

### Clinical characteristics of patients

The basic information and clinical characteristics of patients were summarized in Table 1. The mean age of 104 PNH patients was 42.7 years, with 54% of males. 41% of patients were diagnosed as PNH/aplastic anemia (AA), these patients treated with corticosteroids, blood transfusion supportive therapy, and immunosuppressant for PNH/AA. During the median 719 days of follow-up period, twenty-one thrombotic events occurred in 17 (16.3%) of 104 PNH patients. Only 2 thrombosis occurred at arterial sites and 19 occurred at venous sites (details were shown in Supplementary Table 3). The median time from first diagnosis to thrombotic formation in these 17 patients was 264 days. There have been 4 deaths during the follow-up periods, 2 were attributable to thrombosis, another 2 patients were secondary to the infection and the colorectal carcinoma respectively. Only granulocyte PNH clone sizes was significant higher in thrombus group compared with non-thrombus group (86.24% vs. 59.03%, *P*<0.001), whereas no significant difference was noticed in age, gender, white blood cell and platelet counts, hemolytic parameters, thrombotic parameters (all *P* value>0.05) between the patients with and without thrombosis.

**Table 1:** Clinical characteristics of 104 patients with PNH

Characteristic	Whole cohort	With thrombosis	Without thrombosis	*P* value
Cases, n (%)	104 (100)	17 (16.3)	87 (83.7)	
Age (years)	42.70±15.39	38.94±10.11	43.44±16.16	0.144
Male gender, n (%)	56 (54)	9 (53)	47 (45)	0.935
Bone marrow failure, n (%)	43 (41)	9 (53)	34 (39)	0.289
PNH clone size (%)	63.48±35.53	86.24±19.90	59.03±36.27	**<0.001**
**Blood cells counting**				
Reticulocytes (%)	4.69±3.29	4.12±2.02	4.82±3.51	0.429
Leukocytes (×10^9^/L)	4.01±1.91	4.31±2.28	3.95±1.84	0.49
Hemoglobin (g/L)	79.80±24.55	80.69±18.35	79.64±25.63	0.876
Platelets (×10^9^/L)	109.51±85.55	93.94±98.41	112.44±83.24	0.43
**Hemolytic parameters**				
Lactate dehydrogenase (U/L)	1211.40±883.60	1115.31±713.73	1231.36±917.73	0.635
Total bilirubin (μmol/L)	27.54±21.73	27.68±19.41	27.52±22.27	0.979
Conjugated bilibubin (μmol/L)	7.43±4.23	8.32±3.86	7.25±4.30	0.36
Unconjugated bilibubin (μmol/L)	20.11±19.34	19.36±17.49	20.26±19.79	0.865
Hematuria, n (%)	44 (42)	7 (41)	37 (43)	0.918
**Thrombotic parameters**				
Protein C (%)	111.04±21.01	110.63±27.01	111.12±19.87	0.932
Protein S (%)	105.96±19.91	101.56±25.70	106.80±18.69	0.338
Fibrinogen (g/L)	2.55±0.71	2.79±1.14	2.50±0.59	0.323
D-dimer (mg/L)	0.46±0.52	0.63±0.80	0.43±0.45	0.324
Antithrombin (%)	113.67±14.79	117.38±18.28	112.95±14.05	0.276

*P* value represented the significance of the data between patients with and without thrombosis.

### Association between SNPs and thrombotic risk in PNH

Among the twenty-six alleles in seventeen genes sequenced, nine SNPs (rs8176750, rs146922325, rs1799963, rs1801133, rs397507444, rs199469491, rs199469495, rs121918474, rs6025) were found to have only one genotype. In addition, two SNPs (rs8176704 and rs2289252) did not conform to Hardy-Weinberg equilibrium on statistical analysis (*P*<0.05; shown in Supplementary Table 4). Therefore, these eleven SNPs were excluded for further analysis. Among the rest fifteen SNPs detected, the rs495828 of *ABO* gene was significant relevant to an increased risk of thrombosis in PNH patients, as shown by the dominant model (GT+TT vs. GG: OR 5.95, 95% CI 1.90-18.65, *P*=0.002) and allele model (T vs. G: OR 3.52, 95%CI 1.57-7.90, *P*=0.003). An increased risk of thrombosis was also observed in rs2519093 of *ABO* gene (dominant model: TC+TT vs. CC, OR 6.3, 95% CI 2.01-19.79, *P*=0.001; allele model: T vs. C, OR 3.25, 95%CI 1.43-7.39, *P*=0.006; Table 2). No correlation was found between any other SNPs and thrombotic risk.

**Table 2:** Genotype and allele frequencies of 15 SNPs in PNH patients and their associations with thrombosis

Gene	SNP	Genotype	With thrombosis	Without thrombosis	Dominant model			Recessive model		Allele	With thrombosis	Without thrombosis		
					*P* value	OR (95% CI)	*P* value	OR (95% CI)	*P* value				OR (95% CI)	*P* value
ABO	rs495828	TT	1	1	**0.003**	5.95 (1.90-18.65)	**0.002**	5.38(0.32-90.42)	0.302	T	13	26	3.52 (1.57-7.90)	**0.003**
		TG	11	24						G	21	148		
		GG	5	62										
	rs8176719	CC	6	15	0.087	2.24(0.47-10.63)	0.516	2.62(0.84-8.19)	0.105	C	21	82	1.81 (0.85-3.85)	0.136
		C --	9	52						--.	13	92		
		-- --	2	20										
	rs8176747	GG	0	12	0.242	0.60 (0.21-1.71)	0.428	1.23(1.11-1.35)	0.208	G	7	59	0.51 (0.21-1.23)	0.160
		GC	7	35						C	27	115		
		CC	10	40										
	rs2519093	TT	0	1	**0.002**	6.3 (2.01-19.79)	**0.001**	1.20(1.10-1.31)	1	T	12	25	3.25 (1.43-7.39)	**0.006**
		TC	12	23						C	22	149		
		CC	5	63										
STXBP5	rs1039084	GG	2	9	0.671	1.63 (0.56-4.60)	0.434	1.16(0.23-5.89)	1	G	12	50	1.35 (0.62-2.94)	0.539
		GA	8	32						A	22	124		
		AA	7	46										
vWF	rs1063856	CC	0	1	0.721	1.52 (0.29-8.06)	0.647	1.20(1.10-1.31)	1	C	2	8	1.30 (0.26-6.39)	0.669
		CT	2	6						T	32	166		
		TT	15	80										
GP6	rs1613662	GG	0	0	0.187	3.73 (0.57-24.27)	0.187	--	--	G	2	3	3.56 (0.57-22.19)	0.189
		GA	2	3						A	32	171		
		AA	15	84										
THBD	rs16984852	AA	0	0	1	0.69 (0.03-13.97)	1	--	--	A	0	3	0.71 (0.04-14.07)	1
		AC	0	3						C	34	171		
		CC	17	84										
F11	rs2036914	TT	0	7	0.364	0.54 (0.17-1.66)	0.420	1.21(1.11-1.33)	0.596	T	5	45	0.49 (0.18-1.36)	0.193
		TC	5	31						C	29	129		
		CC	12	49										
FGG	rs2066865	GG	2	24	0.362	0.91 (0.29-2.87)	1	0.35(0.07-1.65)	0.228	G	14	87	0.70 (0.33-1.48)	0.357
		GA	10	39						A	20	87		
		AA	5	24										
SLC44A2	rs2288904	AA	1	10	0.278	1.95 (0.63-6.01)	0.291	0.48(0.06-4.03)	0.687	A	13	58	1.24 (0.58-2.65)	0.693
		AG	11	38						G	21	116		
		GG	5	39										
KNG1	rs710446	CC	1	7	0.950	0.90 (0.31-2.59)	1	0.71(0.08-6.21)	1	C	8	45	0.88 (0.37-2.09)	1
		CT	6	31						T	26	129		
		TT	10	49										
TSPAN15	rs78707713	CC	0	0	0.163	15.91 (0.62-408)	0.163	--	--	C	1	0	15.63 (0.62-392.2)	0.164
		CT	1	0						T	33	174		
		TT	16	87										
PROCR	rs867186	GG	0	0	0.208	2.45 (0.56-10.62)	0.208	--	--	G	3	7	2.31 (0.57-9.42)	0.212
		GA	3	7						A	31	167		
		AA	14	80										
VKORC1	rs9923231	CC	0	0	0.737	1.37 (0.39-4.74)	0.737	--	--	C	4	16	1.32 (0.41-4.21)	0.750
		CT	4	16						T	30	158		
		TT	13	71										

OR: odds ratio; CI: confidence interval

**Figure 1:**
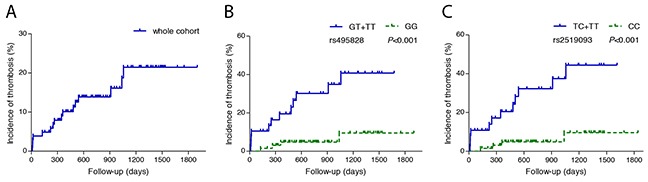
Cumulative incidence of thrombosis in PNH patients with different ABO genotypes **(A)** whole cohort **(B)** thrombosis in different rs495828 genotypes, blue line represent PNH patients with genotype GT and genotype TT, green line represent PNH patients with genotype GG **(C)** thrombosis in different rs2519093 genotypes, blue line represent PNH patients with genotype TT and genotype TC, green line represent PNH patients with genotype CC.

Furthermore, the effect of rs495828/rs2519093 genotype on cumulative incidence of thrombosis was also analyzed, as shown in Figure 1. For rs495828, patients with GT+TT genotype showed significant increased incidence of thrombotic events, compared with patients with GG genotype. Similarly, for rs2519093, TC+TT genotype had higher rate of thrombosis.

### Association between *ABO* haplotypes and thrombotic risk in PNH

**Figure 2:**
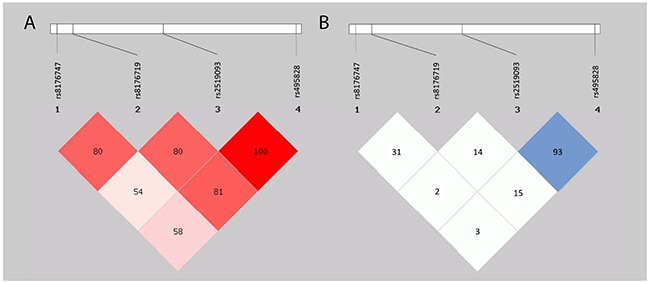
Linkage disequilibrium plots of ABO polymorphisms **(A)** Red squares indicate statistically significant associations between two SNPs which measured by D’; darker shades of red indicate higher D’. **(B)** Blue squares indicate statistically significant associations between two SNPs which measured by r^2^ value; darker shades of blue indicate higher r^2^ value.

In addition to single SNP analysis, haplotype analysis was also performed for the *ABO* gene. rs495828 and rs2519093 were in linkage disequilibrium (LD) (both D’ and r^2^ value ≥80), as shown in Figure 2. Thus, haplotype analysis of these two SNPs was further performed by SHEsis software. As shown in Table 3, the haplotype containing the rs495828 T and the rs2519093 T alleles displayed a significant association with increased risk of thrombosis (OR=3.25, 95%CI 1.43-7.39; *P*=0.004), the haplotype containing the rs495828 G and the rs2519093 C alleles associate with decreased risk of thrombosis (OR=0.28, 95%CI 0.13-0.64; *P*=0.002).

**Table 3:** Haplotypes for the two SNPs in *ABO* gene and their associations with thrombosis risk in PNH patients

rs495828	rs2519093	Case (N=34), n (%)	Control (N=174), n (%)	OR (95%)	*P* value
G	C	21 (62)	148 (85)	0.28 (0.13-0.64)	**0.002**
T	C	1 (3)	1 (1)	5.24 (0.53-51.94)	0.119
T	T	12 (35)	25 (14)	3.25 (1.43-7.39))	**0.004**

OR: odds ratio; CI: confidence interval.

### Analysis of risk factors associated with thrombosis in PNH

In order to identify possible risk factors for thrombosis in PNH, different risk factors like age, gender, rate of bone marrow failure, PNH granulocyte clone size, LDH level, and polymorphism of *ABO* gene were analyzed. Only larger PNH clone and rs495828 GT/TT (or rs2519093 TC/TT) genotype was shown to be related with higher incidence of thrombosis. Multivariate regression analysis showed that larger PNH clone and rs495828 GT/TT (or rs2519093 TC/TT) genotype were independent risk factors for thrombosis in patients with PNH (Table 4).

**Table 4:** Univariate and multivariate regression analysis of risk factors associated with thrombosis in PNH

Parameters	Univariate		Multivariate	
	OR (95% CI)	*P* value	OR (95% CI)	*P* value
**Age**				
≥40 years/<40 year	0.36 (0.12-1.12)	0.077		
**Gender**				
male/female	0.96 (0.34-2.71)	0.935		
**Bone marrow failure**				
with/without	1.75 (0.62-4.99)	0.292		
**PNH granulocyte clone size**			**9.29 (1.14-75.75)^#^**	**0.037^#^**
>50%/≤50%	**9.78 (1.24-77.19)**	**0.031**	9.60 (1.17-78.60)^*^	0.035^*^
**LDH**				
≥1.5 ULN/<1.5 ULN/	2.09 (0.43-10.11)	0.360		
**rs495828**				
GT+TT/GG	**5.63 (1.80-17.60)**	**0.003**	**5.41 (1.67-17.52)^#^**	**0.005^#^**
**rs2519093**				
TC+TT/CC	**5.95 (1.90-18.65)**	**0.002**	5.87 (1.81-19.10)^*^	0.003^*^

OR: odds ratio; CI: confidence interval, ^#^ analyzed PNH clone size and rs495828; ^*^ analyzed PNH clone size and rs2519093.

### Association between *ABO* polymorphism and plasma levels of vWF: Ag and VIII: C

**Figure 3:**
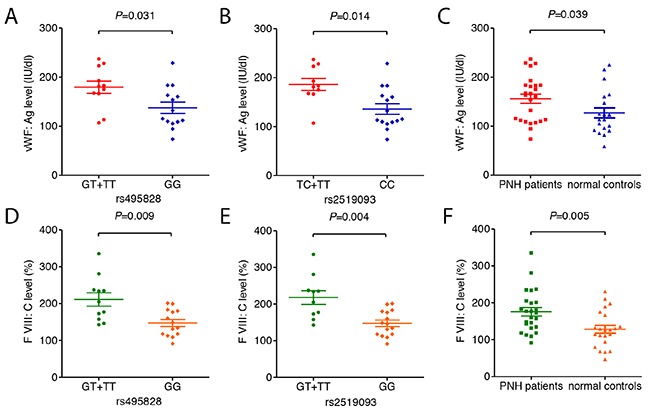
Plasma levels of vWF: Ag and VIII: C **(A)** vWF:Ag between PNH patients with different rs495828 genotypes. **(B)** vWF:Ag between PNH patients with different rs2519093 genotypes. **(C)** vWF:Ag level between PNH patients and normal controls. **(D)** VIII: C level between PNH patients with different rs495828 genotypes. **(E)** VIII: C level between PNH patients with different rs2519093 genotypes. **(F)** VIII: C level between PNH patients and normal controls. Data are presented as mean ± Standard deviation.

Patients carrying rs495828 GT/TT phenotype had a significant higher level of vWF:Ag and VIII: C compared with those carrying rs495828 GG phenotype (vWF:Ag:179±42 IU/dl vs.138±43 IU/dl, *P*=0.031; VIII: C:211±60% vs.148±36%, *P*=0.009, respectively; Figure 3). Similarly, Patients carrying rs2519093 TC+TT phenotype had a significant higher level of vWF:Ag and VIII: C compared with those carrying CC phenotype (vWF:Ag: 186±38 IU/dl vs. 136±42 IU/dl, *P*=0.014; VIII: C: 218±59% vs. 148±35%, *P*=0.004, respectively; Figure 3). Meanwhile, there were no significant correlation between LDH level, PNH clone, platelet counts and plasma levels of vWF:Ag and VIII:C among these patients (Supplementary Figure 1). Further, the PNH cohort had significant higher plasma levels of vWF and factor VIII compared with normal controls (vWF:Ag: 156±47 IU/dl vs. 127±45 IU/dl, *P*=0.039; VIII: C: 176±57% vs. 129±50%, *P*=0.005, respectively; Figure 3).

## DISCUSSION

In our study, 17 (16.3%) of 104 patients were found to have at least one episode of thrombotic event. Although thrombotic rate is much lower in Asian people than in western people, thromboembolism is still the major cause of severe morbidities and early mortality in PNH patients. Several studies have outlined some possible mechanisms which can lead to thrombosis in patients with PNH. Hall *et al.* demonstrated that patients with large PNH granulocyte clone (>50%) had higher thrombotic incidence compared with those with smaller PNH clone, large PNH granulocyte clones are predictive of venous thrombosis [22]. Moyo *et al.* confirmed the association and indicated the risk of thromboembolic events increases by 1.64-fold for every 10% increase in clone size [7]. Lee and colleagues had shown that patients with elevated hemolysis (LDH levels large than 1.5 times the upper limit of normal) at diagnosis were at significant higher risk for thrombosis [8]. However, thrombosis can sometimes occur in patients with small PNH clone, or minor presentations of hemolysis. So there may have some other factors rather than PNH clone size which affect the thromboembolic balance in PNH patients. In some other studies, ethnicity, elevation of D-dimer, transfusion dependence, age more than 55 years were described as risk factors for thrombosis [13, 23, 24], but the results of these risk factors have not been confirmed by other investigators.

To the best of our knowledge, this is the first study to investigate the genetic risk of thromboembolism in patients with PNH. Twenty-six SNPs in 17 genes which had been identified previously to be associated with high risk of venous thromboembolism were selected in this study. These genes encode proteins affect function of coagulation factors, anticoagulation factors, endothelial cells and platelet. The results demonstrated that two SNPs in *ABO* gene, rs495828 and rs2519093, had strong correlation with thrombosis in PNH, the minor alleles of the two SNPs were associated with 5.95 and 6.3 fold increase risk of thrombus formation, compared with major alleles. Haplotype analysis displayed that the combination of twp SNPs could correlation with the thrombosis risk in PNH, an increased risk of thrombosis was observed for the haplotype containing the rs495828 T and the rs2519093 T alleles of the *ABO* gene in PNH patients. Multivariate regression analysis showed that genotype with minor allele of rs495828/rs2519093 and PNH clone size were independent risk factors for thrombosis in PNH. rs2519093, rs495828, rs8176719 in *ABO* gene has been demonstrated to be related with venous thromboembolism by Heit and colleagues [25, 26], but it is the first time to be shown in patients with PNH. Rs495828 locates on the *ABO* exon 7 region, thus affects *ABO* gene expression. While rs2519093 locates on the *ABO* intron 1 region, which does not affect RNA splicing or harbor any suppressor RNA elements, nor is it in linkage disequilibrium (LD) with or closes to known variants in ABO exon 6 and 7 according to previous studies [25, 27]. In this study, the result of haplotype analysis firstly demonstrated rs2519093 were in LD with functional variant (rs495828).

Polymorphism of *ABO* gene could affect the ABO-mediated glycosylation of vWF, thus impact on the plasma levels of vWF and factor VIII. Previous studies had demonstrated that some *ABO* polymorphism correlates with plasma levels ofvWF and factor VIII: people with rs8176719 *ABO* gene deletion (O blood type) have approximately 25% lower level of vWF and factor VIII than those without rs8176719 deletion (non-O blood type) [21, 28]. We then speculated the correlation between the rs495828/rs2519093 polymorphism in *ABO* gene and the levels ofvWF and factor VIII, and found that patients carrying the rs495828/rs2519093 minor allele had an approximately 30% higher level of vWF: Ag and 40% higher level of VIII: C compared with those carrying the major allele. It has been addressed that patients with PNH have higher level of vWF [13], as being demonstrated in our study as well, additionally the level of factor VIII also higher in PNH patients, and higher level of vWF and factor VIII is related with higher risk of thrombosis [29, 30]. Considered the level of vWF and factor VIII may be affected by the function of platelets and endothelial cells, and intravascular hemolysis is the most important factor for the activation of platelets and endothelial cells in PNH [6], we detected the correlation between the PNH clone, LDH level (correlated with intravascular hemolysis), platelet counts, and the level of vWF and factor VIII in patients with PNH, and found no significant correlations (Supplementary Figure 1). In brief, although many other reasons such as endothelial cell activation and damage from hemolysis could also contribute to higher level of vWF and factor VIII, our data strongly suggested that the polymorphism of *ABO* rs495828/rs2519093 probably play a key role, and thus contribute the thrombus formation.

Our findings clearly demonstrate that the rs495828/rs2519093 polymorphism in *ABO* gene represent an independent risk factor in PNH patients for thromboembolism, the mechanism for the associations probably involved in the regulation of plasma level of vWF and factor VIII. However, further studies with larger patient population and longer follow-up duration to verify the conclusions are needed.

## MATERIALS AND METHODS

### Patients

There were totally 104 PNH patients from February, 2011 to August, 2016 in Peking union medical college hospital (PUMCH) enrolled in the study. All patients were confirmed diagnosis through classical symptoms in combination with the proportion of fluorescent aerolysin (FLAER) negative granulocytes higher than 1% [31, 32]. Clinical data including age, sex, clinical symptoms including hemolysis were captured. Laboratory tests including PNH clone size by CD59 and FLAER assay, complete blood count, reticulocyte count, lactate dehydrogenase (LDH) levels, serum bilirubin levels, and thrombophilia risk factors like protein C, protein S, fibrinogen, D-Dimer and antithrombin were detected at the time of the first diagnosis. Thrombotic events were monitored form first diagnosis to December, 2016. This study was performed in accordance with relevant guidelines and was approved by the PUMCH Ethics Committee. Informed consent was obtained from each patient.

### Genotyping

Whole blood samples of 104 patients were collected into tubes containing ethylene diaminetetraacetic acid. Genomic DNA from whole blood was extracted with DNA purification kits (Qiangen, Hilden, Germany). The DNA concentration was measured by spectrometry. Twenty-six SNPs in 17 genes including *MTHFR, PROC, PROS, F2, F5, ABO, Prothrombin* and other genes (shown in Supplementary Table 1) which had been reported as high risk factors were selected for the present study. SNPs were genotyped using the Sequenom MassARRAY system (Sequenom, Inc, San Diego, CA, USA) according to the manufactory protocol. The Primers used for SNPs in this study were listed in Supplementary Table 2.

### Examination of vWF:Ag and VIII:C

Twenty-five PNH patients (11 patients with rs495828 GT/TT genotype, 14 patients with GG genotype) in this study who came back for follow-up were tested for the plasma levels of vWF:Ag and VIII:C through a one stage clotting assay and an enzyme-linked immunosorbent assay (ELISA). Equal number of age and gender matched health were recruited as normal controls and their plasma levels of vWF:Ag and VIII:C were also tested.

### Statistical analysis

Statistical analysis was performed using SPSS 19.0 software. Hardy-Weinberg equilibrium (HWE) was evaluated by comparing expected and observed frequencies using Online Encyclopedia for Genetic Epidemiology HWE tool (OEGE) (http://www.oege.org/software/hwe-mr-calc.shtml) [33]. The observed genotype frequencies were compared to expected values calculated from HWE theory (*p*^2^ + 2*pq* + *q*^2^ = 1; where *p* is the frequency of the major allele and *q* is the frequency of the minor allele) by using the *chi*-squared test. Linkage disequilibrium (LD) and haplotypes analyses were carried out using SHEsis (http://analysis2.bio-x.cn/myAnalysis.php) [34]. Continuous variables were represented as mean ± Standard deviation. Pearson's *chi*-squared test or *fisher* exact test was used to determine whether there was any significant difference in allele and genotype frequencies between PNH patients with or without thrombosis. OR with 95% CI was used to assess the association between the studied SNPs and thromboembolism. Student's *t*-test (data were normal contributed) or Mann-Whitney *U* test (data were not normal contributed) and *fisher* exact test were used to evaluate the correlation between the thrombus formation and the clinical parameters. The influence of genotypes on cumulative incidence of thrombosis was analyzed by the Kaplan-Meier method with log-rank test. Risk factors were identified by univariate and multivariate logistic regression. For all tests, a two-sided *P* value less than 0.05 was considered as statistically significant.

## SUPPLEMENTARY MATERIALS FIGURES AND TABLES


