# Supplementary material for: Polymorphism of the ABO gene associate with thrombosis risk in patients with paroxysmal nocturnal hemoglobinuria
Source: Oncotarget. 2017 Sep 28;8(54):92411–9. doi: 10.18632/oncotarget.21361 (PMC5696192; doi:10.18632/oncotarget.21361)
Supplement: Supplementary file 1 [file oncotarget-08-92411-s001.pdf]

Polymorphism of the *ABO* gene associate with thrombosis risk in patients with paroxysmal nocturnal hemoglobinuria

SUPPLEMENTARY MATERIALS

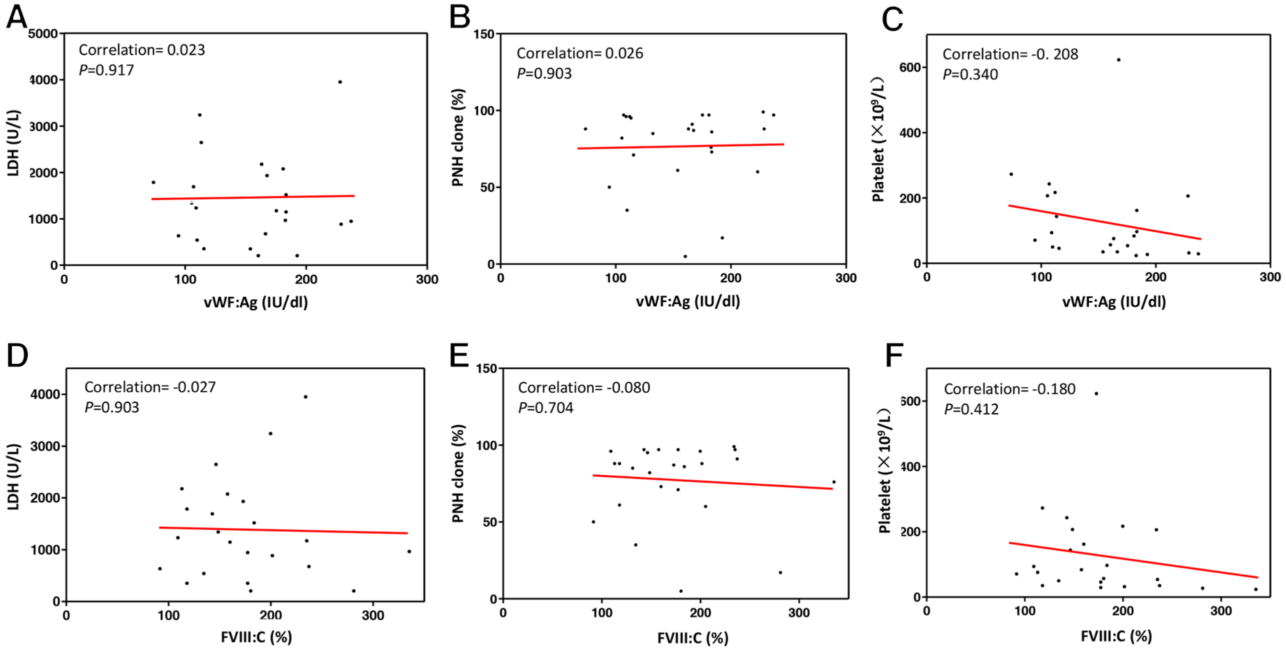

Supplementary Figure 1: Correlation with the level of vWF:Ag and FVIII: C with possible factors among PNH patients: Correlation of the level of vWF:Ag with LDH **(A)**, PNH clone **(B)**, platelet **(C)**; correlation of the level of FVIII:C with LDH **(D)**, PNH clone **(E)**, platelet **(F)**.

Supplementary Table 1: Twenty-six alleles of 17 genes were detected in this study

Gene name	SNP	Associated phenotype
**ABO**	rs495828	vWF↑, VIII↑
	rs8176704	vWF↑, VIII↑
	rs8176719	vWF↑, VIII↑
	rs8176747	vWF↑, VIII↑
	rs8176750	vWF↑, VIII↑
	rs2519093	vWF↑, VIII↑
**STXBP5**	rs1039084	vWF↑
**vWF**	rs1063856	vWF↑
**PROC**	rs146922325	Protein C↓
**GP6**	rs1613662	platelet activation
**THBD**	rs16984852	thrombomudulin↓
**F2**	rs1799963	FII↑
**MTHFR**	rs1801133	methylenetetrahydrofolate reductase↓
	rs397507444	methylenetetrahydrofolate reductase↓
**PROS1**	rs199469491	Protein S↓
	rs199469495	Protein S↓
	rs121918474	Protein S↓
**F11**	rs2036914	FXI↑
	rs2289252	FXI↑
**FGG**	rs2066865	Fibrinogen YY'↓
**SLC44A2**	rs2288904	unkonwn
**F5 leiden**	rs6025	Resistance to activated pro C
**KNG1**	rs710446	aPTT↓
**TSPAN15**	rs78707713	unkonwn
**PROCR**	rs867186	sEPCR↑
**VKORC1**	rs9923231	vitamin K↑

Supplementary Table 2: Primers used for this study

SNP	1st-PCRP	2nd-PCRP	UEP_SEQ
**rs495828**	ACGTTGGATGCAGGTGTCATGGAGAAATGG	ACGTTGGATGACCTGTAACTGTTGCAAGGG	TTGCAAGGGAGGTAAA
**rs8176704**	ACGTTGGATGATTCACCAGACACGTCGAAG	ACGTTGGATGGGGCCTCATGGTTTGTAGAC	AAACTCCACTCTGCC
**rs8176719**	ACGTTGGATGTGTCGATGTTGAATGTGCCC	ACGTTGGATGCATGTGCAGTAGGAAGGATG	AGGATGTCCTCGTGGT
**rs8176747**	ACGTTGGATGACGAGGGCGATTTCTACTAC	ACGTTGGATGTGGTCGACCATCATGGCCTG	ACCGACCCCCCGAAGAAC
**rs8176750**	ACGTTGGATGTAAAACCAAGGGCGGGAGG	ACGTTGGATGGGTGCCCAAGAACCACCAG	AGGCGGTCCGGAACC
**rs2519093**	ACGTTGGATGTATACACCTACTATGAACCC	ACGTTGGATGAATGAATAAATAAGCCACC	cccccTAAGCCACCGACTGA
**rs1039084**	ACGTTGGATGGGAGGTATGTAGTATGCTTG	ACGTTGGATGTAACTTTGAGCACCCAAGCC	AAGCCCCAATTACCTCCG
**rs1063856**	ACGTTGGATGCATGGTCAAGCTGGTGTGTC	ACGTTGGATGTGCACTCCAGGTCATAGTTC	TCTGGCACGTTTTGG
**rs146922325**	ACGTTGGATGGGTCTTCTGTGTCTCGTTTC	ACGTTGGATGCTCAGTGAAGTTCCCTTGTG	ccgGGGAGGCCCTGGAAG
**rs1613662**	ACGTTGGATGATTTCCCAGGAACCTCTGTG	ACGTTGGATGATGGACCCTGCAGAACCTAC	tcACCTACCTGCTACCG
**rs16984852**	ACGTTGGATGGCACTTATAAACTCGAGCCC	ACGTTGGATGAGCCCAGACACTTCTTGCC	ctcaGCTGCGCGCAGCCCCTG
**rs1799963**	ACGTTGGATGTGAATAGCACTGGGAGCATT	ACGTTGGATGTGGAACCAATCCCGTGAAAG	gttcaCAATAAAAGTGACTCTCAGC
**rs1801133**	ACGTTGGATGCTTGAAGGAGAAGGTGTCTG	ACGTTGGATGCTTCACAAAGCGGAAGAATG	gaagGCTGCGTGATGATGAAATCG
**rs397507444**	ACGTTGGATGGCTGACCAGTGAAGAAAGTG	ACGTTGGATGCTCACTTTGTGACCATTCCG	tAGAGGTAAAGAACGAAGACT
**rs199469491**	ACGTTGGATGCACCTCCAGTTGTGATTTTG	ACGTTGGATGTTGCACTTCGTGGTGGAAAG	AAGATTGAAGTTCAGCTTAAGAA
**rs199469495**	ACGTTGGATGAGCTAAAGAAGCTGTGATGG	ACGTTGGATGACTTTGGTTTCCAGCAATCC	aGGCTTAAAAAGGGGTCCA
**rs121918474**	ACGTTGGATGTCTACCATCCTGCTCTTACC	ACGTTGGATGGGAAGTTACCACTGTTCCTG	aaagGGTTTTGTTATGCTTTCAAAT
**rs2036914**	ACGTTGGATGTGTCTTGGAGACAAGGAGTG	ACGTTGGATGAAAGCAGCAGCTTTTGCCAG	AGCAGCTTTTGCCAGTAAAGA
**rs2289252**	ACGTTGGATGTTTTCCTCCCTCAGGTTCAG	ACGTTGGATGAGTTGGATGAGGAGTTAGCG	GTGAGGCTTGTCTCTCTCT
**rs2066865**	ACGTTGGATGATTACTACTGATGGTTGCCC	ACGTTGGATGGTTTCCTAAGACTAGATAC	TTCCTAAGACTAGATACATGGTA
**rs2288904**	ACGTTGGATGATCTCTCTTGGCAGGGAGTG	ACGTTGGATGTGACTCACAGGGTTTGCTGG	GGGCAGTCACCATCT
**rs6025**	ACGTTGGATGCTCTGGGCTAATAGGACTAC	ACGTTGGATGCTTCAAGGACAAAATACCTG	AAGGACAAAATACCTGTATTCCT
**rs710446**	ACGTTGGATGTCAGGGATCCAATCGTCATC	ACGTTGGATGCAGGACTCTGATCTCATTGC	ATATCACCAGCTCCCA
**rs78707713**	ACGTTGGATGTTCGACTTCACCTTCCACCC	ACGTTGGATGGGAAGCTGAGCAGAAATGAG	cTGTGAGCAAAACAGCG
**rs867186**	ACGTTGGATGACAAGCCGCTCCTACACTTC	ACGTTGGATGAAGATGCCTACAGCCACACC	ACCAGCAATGATGAAAC
**rs9923231**	ACGTTGGATGTCTGGGAAGTCAAGCAAGAG	ACGTTGGATGGCTAGGATTATAGGCGTGAG	aggaGTGAGCCACCGCACC

Supplementary Table 3: Sites of thrombosis in 17 patients

Thrombus site	Events, n	% of total
Venous		
Portal vein	6	28.6
Mesenteric vein	3	14.3
Cerebral vein	3	14.3
Splenic vein	3	14.3
Deep vein	3	14.3
Pulmonary embolus	1	4.8
Arterial		
Cornary	2	9.5
Total	21	100

Supplementary Table 4: Hardy-Weinberg equilibrium and minor allele frequency of 17 SNPs

Gene name	SNP	x^2	*P* value	MAF	MAF in database
ABO	rs495828	1.136	0.286	0.1875	0.1603
	rs8176704	104	<0.001	0.0577	0.0463
	rs8176719	3.119	0.077	0.4952	0.3438
	rs8176747	0.479	0.489	0.3173	0.1528
	rs2519093	2.36	0.125	0.1779	0.1414
STXBP5	rs1039084	0.68	0.41	0.2981	0.4571
vWF	rs1063856	2.649	0.104	0.0481	0.3363
GP6	rs1613662	0.063	0.802	0.024	0.1573
THBD	rs16984852	0.022	0.881	0.0144	0.0014
F11	rs2036914	0.283	0.595	0.2356	0.3936
	rs2289252	6.481	0.011	0.2596	0.3195
FGG	rs2066865	0.337	0.562	0.4856	0.3027
SLC44A2	rs2288904	0.238	0.626	0.3413	0.1827
KNG1	rs710446	0.415	0.519	0.2548	0.4153
TSPAN15	rs78707713	0.002	0.96	0.0048	0.0479
PROCR	rs867186	0.265	0.607	0.0481	0.096
VKORC1	rs9923231	1.177	0.278	0.0962	0.3556
